# Demineralized Cortical Bone Matrix Augmented With Peripheral Blood-Derived Mesenchymal Stem Cells for Rabbit Medial Meniscal Reconstruction

**DOI:** 10.3389/fbioe.2022.855103

**Published:** 2022-04-27

**Authors:** Beini Mao, Zhong Zhang, Sike Lai, Kaibo Zhang, Jian Li, Weili Fu

**Affiliations:** ^1^ Department of Orthopedics, Orthopedic Research Institute, West China Hospital, Sichuan University, Chengdu, China; ^2^ Department of Orthopaedics, No.3 People’s Hospital of Chengdu, Chengdu, China

**Keywords:** sports medicine, meniscus, peripheral blood-derived mesenchymal stem cells, demineralized cortical bone matrix, tissue-engineer

## Abstract

Tissue engineering is a promising treatment strategy for meniscal regeneration after meniscal injury. However, existing scaffold materials and seed cells still have many disadvantages. The objective of the present study is to explore the feasibility of peripheral blood-derived mesenchymal stem cells (PBMSCs) augmented with demineralized cortical bone matrix (DCBM) pretreated with TGF-β3 as a tissue-engineered meniscus graft and the repair effect. PBMSCs were collected from rabbit peripheral blood and subjected to three-lineage differentiation and flow cytometry identification. DCBM was prepared by decalcification, decellularization, and cross-linking rabbit cortical bone. Various characteristics such as biomechanical properties, histological characteristics, microstructure and DNA content were characterized. The cytotoxicity and the effects of DCBM on the adhesion and migration of PBMSCs were evaluated separately. The meniscus-forming ability of PBMSCs/DCBM complex *in vitro* induced by TGF-β3 was also evaluated at the molecular and genetic levels, respectively. Eventually, the present study evaluated the repair effect and cartilage protection effect of PBMSCs/DCBM as a meniscal graft in a rabbit model of medial meniscal reconstruction in 3 and 6 months. The results showed PBMSCs positively express CD29 and CD44, negatively express CD34 and CD45, and have three-lineage differentiation ability, thus can be used as tissue engineering meniscus seed cells. After the sample procedure, the cell and DNA contents of DCBM decreased, the tensile modulus did not decrease significantly, and the DCBM had a pore structure and no obvious cytotoxicity. PBMSCs could adhere and grow on the scaffold. Under induction of TGF-β3, PBMSCs/DCBM composites expressed glycosaminoglycan (GAG), and the related gene expression also increased. The results of the *in vivo* experiments that the PBMSCs/DCBM group had a better repair effect than the DCBM group and the control group at both 12 and 24 weeks, and the protective effect on cartilage was also better. Therefore, the application of DCBM augmented with PBMSCs for meniscus injury treatment is a preferred option for tissue-engineered meniscus.

## 1 Introduction

The meniscus is wedge-shaped fibrocartilage between the femoral condyle and tibial plateau. The superior load transmission and shock absorption capability avoid excessive stress on the articular cartilage, thus enhancing joint stability (Walker and Erkman, 1975; Walker et al., 2015). However, the meniscus is easily damaged for sports-associated activities, trauma or age-related complications (Gee et al., 2020; Rhim et al., 2021). The current well-established treatment, partial or total meniscectomy, could not achieve the biological healing of the meniscus and might be related to knee osteoarthritis (OA) (Cross et al., 2014; Kwon et al., 2019). Therefore, it is necessary to explore the biological treatment of meniscus injuries. Cell-based tissue engineering and regenerative medicine strategies have been advocated as a potential approach to address this issue (Rhim et al., 2021).

Mesenchymal stem cells (MSCs) are immature, unspecialized cells that can be isolated from adult bone marrow, adipose tissue, blood, umbilical cord, skeletal, muscle, dental pulp, deciduous teeth, and periodontal ligaments. MSCs have been widely used as seed cells in meniscus tissue engineering. The repair effect of bone marrow-derived MSCs (BM-MSCs), synovium-derived MSCs (S-MSCs), and adipose-derived MSCs (A-MSCs) have been proved by plenty of studies (Zellner et al., 2017; Onoi et al., 2019; Ozeki et al., 2021). However, problems such as difficulty obtaining and pain in the donor site may affect their clinical application. Peripheral blood-derived MSCs (PBMSCs) can be harvested from peripheral blood, and this unique advantage in the collection gives it potential in a clinical application (Calle et al., 2021). However, there has been limited information about PBMSCs used in meniscal reconstruction.

Demineralized bone matrix (DBM) is prepared from bone matrix and consists of a collagen scaffold containing several growth factors, such as bone morphogenetic proteins (BMPs), insulin growth factor, transforming growth factor, and fibroblast growth factor (Urist, 1965). It has been widely used in bone tissue engineering as a natural tissue-derived scaffold material. In recent years, studies have also been using DBM for soft tissue repair (Yamada, 2004), including meniscus (Zhang et al., 2015; Yuan et al., 2016). However, the osteoinductive characteristics limited its further application on meniscus repair (Zhou et al., 2012). In addition, the previous studies all used demineralized cancellous bone, which was worse than the demineralized cortical bone matrix (DCBM) in terms of mechanical properties. DCBM has been widely used in the cartilage repair and tendon-bone healing field (Gao et al., 2004; Thangarajah et al., 2017; Thangarajah et al., 2018). While there has been limited researches using it in meniscal reconstruction to our best knowledge.

Transforming growth factor-β3 (TGF-β3) is one of the main members of the TGF family of cytokines, promoting the proliferation and differentiation of chondrocytes, promoting the formation of extracellular matrix, and inhibiting various inflammatory processes cytokines such as IL-1, MMPs, and TNF-α. It plays an important role in wound repair, especially in the growth and reconstruction of cartilage (Bian et al., 2011).

Based on the current limitation of tissue engineering in meniscus regeneration, the objective of the present study is to explore the treatment effect of demineralized cortical bone matrix scaffold augmented with PBMSCs pretreated with TGF-β3 in meniscus injury.

## 2 Materials and Methods

### 2.1 Cell Isolation and Culture

#### 2.1.1 Isolation of Peripheral Blood-Derived Mesenchymal Stem Cells

PBMSCs were isolated from 3-month-old New Zealand white rabbits. As previously reported, this procedure was performed (Fu et al., 2015). Briefly, rabbits were pretreated with granulocyte colony-stimulating factor (G-CSF) once daily for 5 days (50 μg/kg i.h.) and AMD3100 was administered on the sixth day (5 mg/kg i.h.) 1 h before blood sampling. The blood samples were harvested using a sterile syringe from the central ear artery and transformed into a centrifugal tube. The nucleated cells were isolated by density gradient centrifugation, seeded in a 10 cm dish at a density of 10^6^ cells/mL, and cultured with a complete medium consisting of low-glucose Dulbecco’s modified Eagle’s medium (DMEM) and 16% fetal bovine serum (FBS). After 10–14 days, colonies were formed. In this study, we use polyclonal PBMSCs mixed from multiple clones. PBMSCs at the third passage were utilized for further experiments. Crystal violet staining was used to assess colony formation.

#### 2.1.2 Multipotent Differentiation of Peripheral Blood-Derived Mesenchymal Stem Cells

The multipotent differentiation potential of PBMSCs was evaluated through adipogenic, osteogenic, and chondrogenic induction *in vitro*, as previous reported (Fu et al., 2014). Adipogenesis, osteogenesis, and chondrogenesis differentiation were validated by Oil red O staining, alizarin red staining, and alcian blue staining, respectively.

#### 2.1.3 Immunophenotypic Identiﬁcation of Mesenchymal Stem Cells by Flow Cytometry

The surface markers of PBMSCs at passage 3 were analyzed using flow cytometry (FCM). Accordingly, MSCs were harvested with trypsin/EDTA and then incubated with CD29, CD34, CD44, and CD45 primary antibodies, respectively, for 30 min, followed by secondary fluorescein antibody for 30 min. Finally, cells were fixed in flow buffer, washed, and suspended in 0.2 ml of PBS for FCM analysis using Cell Quest software (BD Biosciences, San Jose, California). Parallel blank tubes were used as controls.

### 2.2 Preparation of Demineralized Cortical Bone Matrix Scaffolds

The DCBM scaffolds were prepared according to the protocols of previous studies (Fu et al., 2014). Cortical bone was obtained from the midshafts of the femur and tibia of adult New Zealand white rabbits and DCBM was subjected to the following procedures: 1) demineralization in 0.6 mol/L hydrochloric acid at 4°C for 7 days, 2) defatting under 1:1 (vol/vol) methanol/chloroform solution for 24 h, and 3) deproteinization with 3% hydrogen peroxide for 12 h 4) shake in 1% Triton X-100 and 0.5% NaDC solution for 24 h, and wash with PBS 3 times. Then incubate with nuclease solution (150 IU/ml DNase and 100 μg/ml RNase) at 37°C for 24 h with shaking. 5) Crosslink using ethyl dimethylaminopropyl carbodiimide (0.6 mol/L EDAC; Sigma-Aldrich) in a crescent-shaped mold to form the DCBM scaffold.

### 2.3 Characterization of Scaffold

#### 2.3.1 Histological Evaluation

After being fixed with 4% paraformaldehyde for 24 h, the DCBM was dehydrated with gradient alcohol. Then it underwent wax dipping and embedding. The wax block with samples encapsuled was then cut into 4 μm sections. After dewaxing, the section was stained with hematoxylin for 5 min and eosin for 3 min, as HE staining. And another section was soaked in hematoxylin for 5 min, ponceau for 5 min, 2% glacial acetic acid for a while, 2.5% phosphomolybdic acid aqueous for 5 min and aniline blue for 5 min, as Masson staining.

#### 2.3.2 Microstructure of Scaffold

The surface and section microstructure of DCBM was characterized by SEM. Before SEM imaging, dried samples were cut with a razor blade in liquid nitrogen and sputter-coated with platinum. SEM was used to observe the microstructure of the surfaces and sections of the samples at an accelerating voltage of 15 kV.

#### 2.3.3 DNA Content Assay

Ten mg of sample was cut into pieces, digested with 1 ml of metalloproteinase K (0.2 mg/ml) at 60°C for 24 h, and centrifuged at 15 kg for 30 min. The DNA content assay was performed according to the instructions of the kit (Quant-iTTM PicoGreen^®^ ds DNA Assay Kit), and a cortical bone that was not undecellularized was set as a control group.

#### 2.3.4 Biomechanical Testing

An Instron (Norwood, MA) 5,543 testing frame was utilized to analyze the tensile properties of the hydrated constructs. Testing samples (*n* = 7) were hydrated for at least 30 min prior to testing. The specimen was fixed with a specific fixture, loaded on a biomechanical testing instrument, preloaded with 0.1 N, loaded at a rate of 0.5 mm/s and recorded the stress-strain curve to calculate the tensile elasticity modulus. The normal meniscus of rabbits was used as the control group.

### 2.4 *In Vitro* Studies

#### 2.4.1 Cytotoxicity of the Scaffold

To evaluate the cytotoxicity of the scaffold to PBMSCs, CCK-8 was used. A certain quality of DCBM scaffold was weighed and cut into pieces. After sterilization with ethylene oxide, the scaffold was added into a 6-well plate, as well as 10 times the quality of serum-free medium was added. The supernatant was collected as an extract medium 3 days later. The extract medium of the scaffold was used to culture the PBMSCs in a 96-well plate, as well as the normal medium was used to culture the same batch of cells as control. At the end of culture, 110 μl of CCK-8 working solution (volume ratio CCK-8: DMEM = 1:10) was added to each well after 2-h incubating at 37°C, the OD value of 450 nm was detected in a microplate reader.

#### 2.4.2 Live/Dead Staining

Before seeding with cells, scaffolds were sterilized, washed in sterile PBS, and treated with DMEM overnight. Each scaffold was seeded with 10 μl of 1.2*10^4^/μl PBMSCs every 10 min for 4 times. Cells were allowed to adhere for 4 h and then the scaffolds were transferred to the incubator and the medium was changed every day.

To evaluate the viability of PBMSCs seeded in the material, a Live/Dead Assay kit (Invitrogen) was used. The cell/scaffold constructs were washed with sterile PBS and incubated in PBS solution with 2 mM calcein AM and 4 mM ethidium homodimer-1 for 1 h at room temperature, according to the instructions of the kit. After another wash with sterile PBS, constructs were observed using a confocal microscope (Leica).

#### 2.4.3 Cell Adhesion

We utilize the SEM to observe the microstructure of the cell-scaffold composites and the adhesion of PBMSCs cultured *in vitro* on the scaffolds. Cell-scaffold composites were acquired in 1, 2 and 3 days after seeding the cells. Samples were fixed in 2.5% (v/v) glutaraldehyde for 24 h, washed with PBS, dehydrated in graded alcohol and dried with CO_2_ critical point drying. After coating with gold, cell-scaffold composites were observed using the SEM.

#### 2.4.4 Cell Migration

PBMSCs of passage 3 were seeded in a 6-well plate. Moreover, when the cells were more than 90% confluent, they were replaced with serum-free starvation for 12 h. A 200 μL gun tip was used to scratch along the straight line drawn in advance; then, the scratched cells were washed by PBS. A DCBM extract medium was used to culture the PBMSCs, while the normal medium was used in the control group. In addition, to see the effect of TGF-β3 on cell behaviors, a DCBM extract + TGF-β3 (concentration) group was set, in which 10 ng/ml TGF-β3 was added into the extract medium. All scratches were observed using a microscope at 0, 8, and 24 h after the intervention.

#### 2.4.5 Biochemical Assays for Glycosaminoglycan

In order to evaluate the effect of DCBM on the differentiation of PBMSCs and the induction ability of TGF-β3 at the molecule level, we detected the deference of GAG content. After seeding, the cell-scaffold composites in two experimental groups were cultured in DMEM or DMEM with TGF-β3 (10 ng/ml), while an equal quantity of PBMSCs was seeding in a 12-well plate in the control group and DMEM was used to culture it. The mediums were changed every 2 days. After 3, 7 and 14 days of culture, the GAG in the scaffolds of the 3 groups were evaluated according to the GENMED quantitative detection kit. A previous study described the method and principle in detail (Barbosa et al., 2003).

#### 2.4.6 Cartilage-Related Gene Expression Analysis

To evaluate the effect of DCBM on the differentiation of PBMSCs and the induction ability of TGF-β3 at the gene level, we decided to detect the expression level of a cartilage-related gene using polymerase chain reaction (PCR). The experimental grouping and culture are the same as in biochemical assays for GAG. After 3, 7 and 14 days of culture, the cells were harvested, washed with PBS and blew with 1 ml Trizol for 5 min. After adding 200 μl of chloroform, shaking for 15 s, the liquid was centrifuged in 14 kg for 15 min. After centrifugation, 400 μl liquid in the upper aqueous layer was carefully aspirated and mixed with 400 μl isopropanol. After centrifuging the sample at 14 kg for 10 min 75% alcohol was used to dissolve the white feather-like RNA precipitation adhered to the bottom wall of the EP tube. After another centrifugation at 15 kg for 5 min, DEPC water was used to dissolve the RNA.

Reverse transcription was performed using Promega’s GoScript Reverse Transcription system and Bio-Rad’s DNA Engine PCR machine. Moreover, amplification was performed using a Roche LightCycler96 PCR instrument.

The detected gene and primer sequence are listed in Table 1 **.**

**TABLE 1:** Gene and primer sequence.

Genes		Primer nucleotide sequence
SOX9	Forward	GCG​GAG​GAA​GTC​GGT​GAA​GAA​T
	Reverse	AAG​ATG​GCG​TTG​GGC​GAG​AT
Aggrecan	Forward	GTG​AAA​GGT​GTT​GTG​TTC​CAC
	Reverse	TGG​GGT​ACC​TGA​CAG​TCT​GAT
Coll I	Forward	GCC​ACC​TGC​CAG​TCT​TTA​CA
	Reverse	CCA​TCA​TCA​CCA​TCT​CTG​CCT
Coll Ⅱ	Forward	CAC​GCT​CAA​GTC​CCT​CAA​CA
	Reverse	TCT​ATC​CAG​TAG​TCA​CCG​CTC​T
Coll Ⅲ	Forward	GAG​CCT​CCC​AGA​ACA​TCA​CC
	Reverse	GTA​GTC​TCA​CAG​CCT​TGC​GT
GAPDH	Forward	CAA​GAA​GGT​GGT​GAA​GCA​GG
	Reverse	CAC​TGT​TGA​AGT​CGC​AGG​AG

### 2.5 *In Vivo* Animal Studies

#### 2.5.1 Graft Preparation

The preparation procedure is described in 2.2. After sterilization, the scaffold was washed in sterile PBS, and immersed in DMEM overnight. Then 10 μl of 2.5×10^4^/μl PBMSCs suspension was added to the DCBM scaffold every 10 min for 4 times. Cells were allowed to adhere for 4 h, and then, medium with 16% FBS and 10 ng/ml TGF-β3 was added to the plate. The plate was transferred to an incubator and cultured for 1 week, and the medium was changed every 2 days.

#### 2.5.2 Surgical Procedure

Thirty-two adult New Zealand white rabbits weighing about 2.5 kg were selected. After the medial meniscus was removed, the DCBM + PBMSCs complex and DCBM scaffolds were implanted *in situ* in right and left knee, respectively, in 16 rabbits. The rest 16 rabbits were treated with incision of the skin and joint capsule, but not the meniscus, in the right knee, as the sham-operated group, and the left knee with the meniscus removed as the control group. The cell inoculation method was the same as mentioned above. The amount of PBMSCs seeded on a single material is 10^6^. After cell seeding, the complex was cultured in DMEM with 16% FBS and 10 ng/ml TGF-β3 for 1 week and then implanted.

**FIGURE 1:**
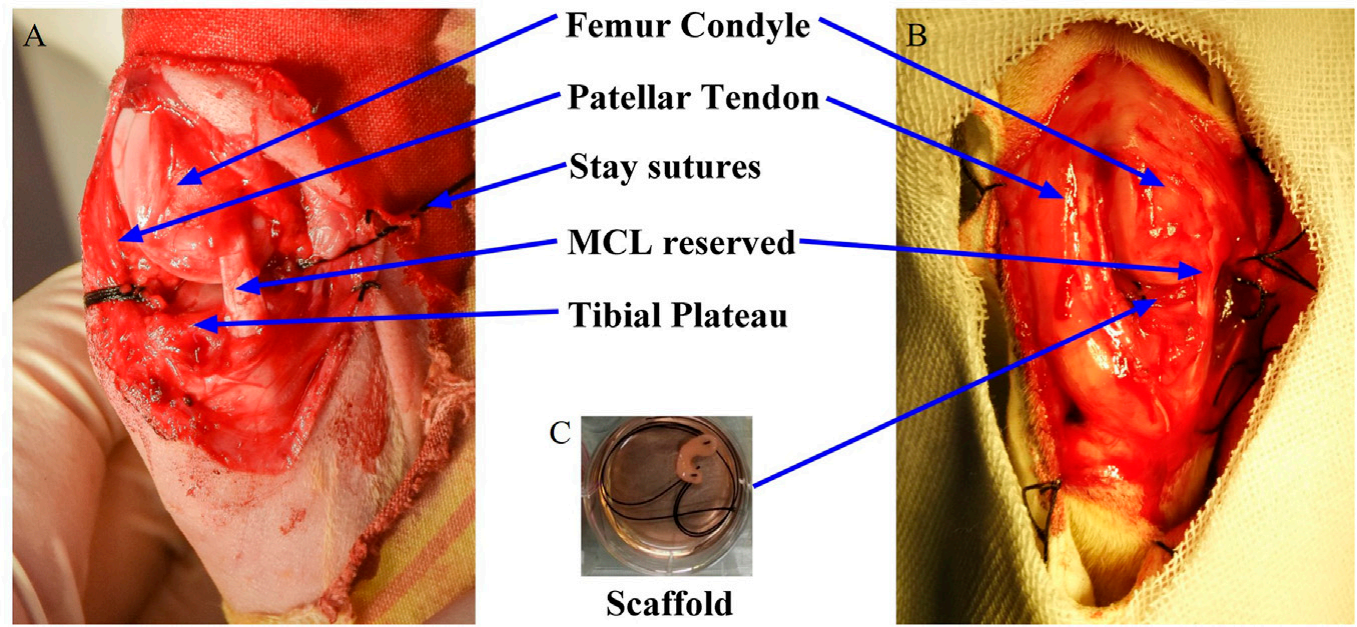
Intraoperative pictures of meniscus graft implanted into knee joint and surgery grouping. **(A)** removal of meniscus; **(B)** graft implanted; **(C)** meniscus graft.

After anesthesia and routine preparation, the knee was approached through a medial parapatellar incision. A total meniscectomy was performed by resecting the medial meniscus sharply along the periphery and detaching it from its anterior and posterior junction. The medial collateral ligament, which is important for the postoperative stability of the knee joint, was reserved. Both the anterior and posterior horns and periphery of the scaffolds were reattached to the respective root attachments and appropriate adjacent synovium with absorbable No. 4-0 sutures. For the posterior root, attachment of the medial meniscus is adjacent to the posterior cruciate ligament. A self-made threading apparatus and extracapsular knot-ting technique were used to fix the posterior horn of the scaffold with the ligamentous structures. The joint capsule, periarticular tissue, and skin were closed with No. 3-0 Vicryl sutures. Figure 1A showed the picture of rabbits’ knee after meniscus removal, Figure 1B showed the pictures of rabbits’ knee after the meniscus graft implanted and Figure 1C showed the meniscus graft.

#### 2.5.3 Evaluation of Implants

The animals were sacrificed at 12 and 24 weeks after the operation. The femur and tibia of the knee joint were dissected out, and the newly formed meniscus tissue was freed and photographed.

Nine aspects of meniscus fusion, position, anterior and posterior angle position, size, tear, surface, shape, tissue, and synovium were evaluated and scored (Chiari et al., 2006). A higher score indicated a worse prognosis.

In addition, the newly formed meniscus was prepared as a pathological section according to conventional procedures. HE staining, toluidine blue staining, safranin O staining, Sirius red staining, MASSON staining, and immunohistochemical staining of collagen types I and II were performed on the meniscus, and the semi-quantitative histological evaluation of the meniscus-like tissue was performed using the Ishida score (Ishida et al., 2007).

#### 2.5.4 Evaluation of Cartilage

Similar to the meniscus, the cartilage was also evaluated in gross evaluation through International Cartilage Repair Society (ICRS) cartilage lesion classification (Mainil-Varlet et al., 2003) and in histology through the Mankin grading system (Baklaushev et al., 2019).

### 2.6 Statistical Analysis

The data are expressed as mean ± standard deviation. SPSS 16.0 (IBM, Armonk, New York) was used to conduct the analysis. The independent samples t test was used for comparison between two groups, and the one-way ANOVA test was used for comparison between multiple groups and LSD was used for post hoc test. Differences were considered significant at *p* < 0.05.

## 3 Results

### 3.1 Cell Culture and Identiﬁcation

#### 3.1.1 Cell Culture

**FIGURE 2:**
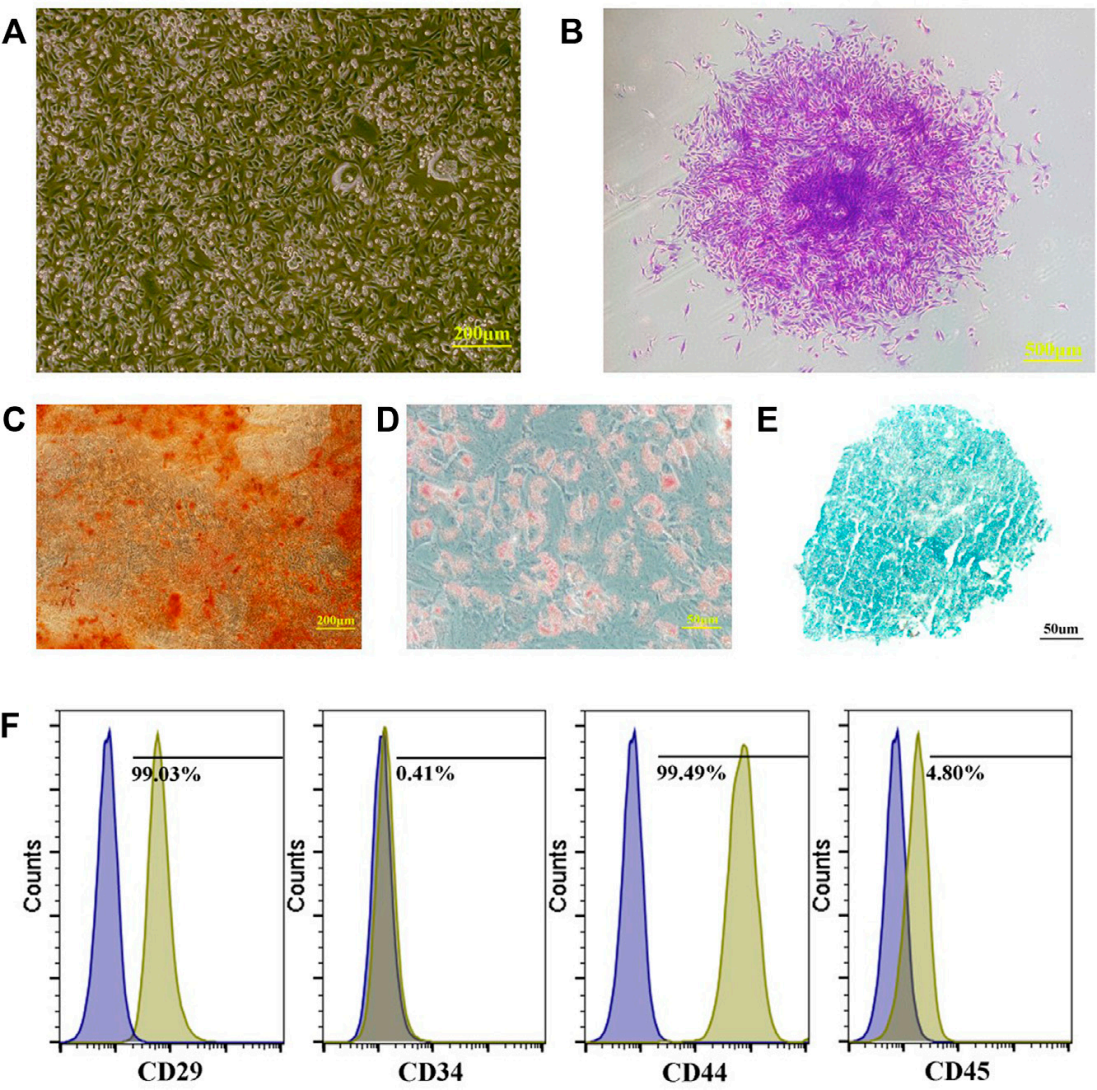
Preparation and identification of PBMSCs. **(A)** primary cells of PBMSCs; **(B)** apparent colonies stained with crystal violet; **(C)** PBMSCs stained with alizarin red after osteogenic induction; **(D)** PBMSCs stained with oil red O after adipogenic induction; **(E)** PBMSCs stained with alician blue after chondrogenic induction and **(F)** immunophenotypic characterization of PBMSCs.

Adherent cells were observed after 5–7 days of primary cell culture. Among them, the spindle-shaped cells proliferated relatively quickly. After culturing for about 2 weeks, almost all cells were spindle-shaped, and they moved after 10–15 days. The colonies were formed and the cell morphology was uniformly spindle-shaped with time going by (Figures 2A,B).

#### 3.1.2 Multipotent Differentiation of Peripheral Blood-Derived Mesenchymal Stem Cells

Many calcium nodules were observed in osteogenic induced cells after alizarin red staining (Figure 2C). Oil red O staining showed that lipid-rich vesicles are formed in the cells after adipogenic differentiation (Figure 2D). Alcian blue staining showed a blue cell sphere after chondrogenic induction (Figure 2E). The results showed that PBMSCs could perform osteogenic, adipogenic, and chondrogenic differentiation under induction.

#### 3.1.3 Immunophenotypic Identiﬁcation of Mesenchymal Stem Cells by Flow Cytometry

Flow cytometry analysis showed that the PBMSCs had a positive expression of CD29 and CD44 and a negative expression of CD34 and CD45 (Figure 2F).

### 3.2 Characterization of the Demineralized Cortical Bone Matrix Scaffold

#### 3.2.1 Histological Evaluation of the Demineralized Cortical Bone Matrix Scaffold

**FIGURE 3:**
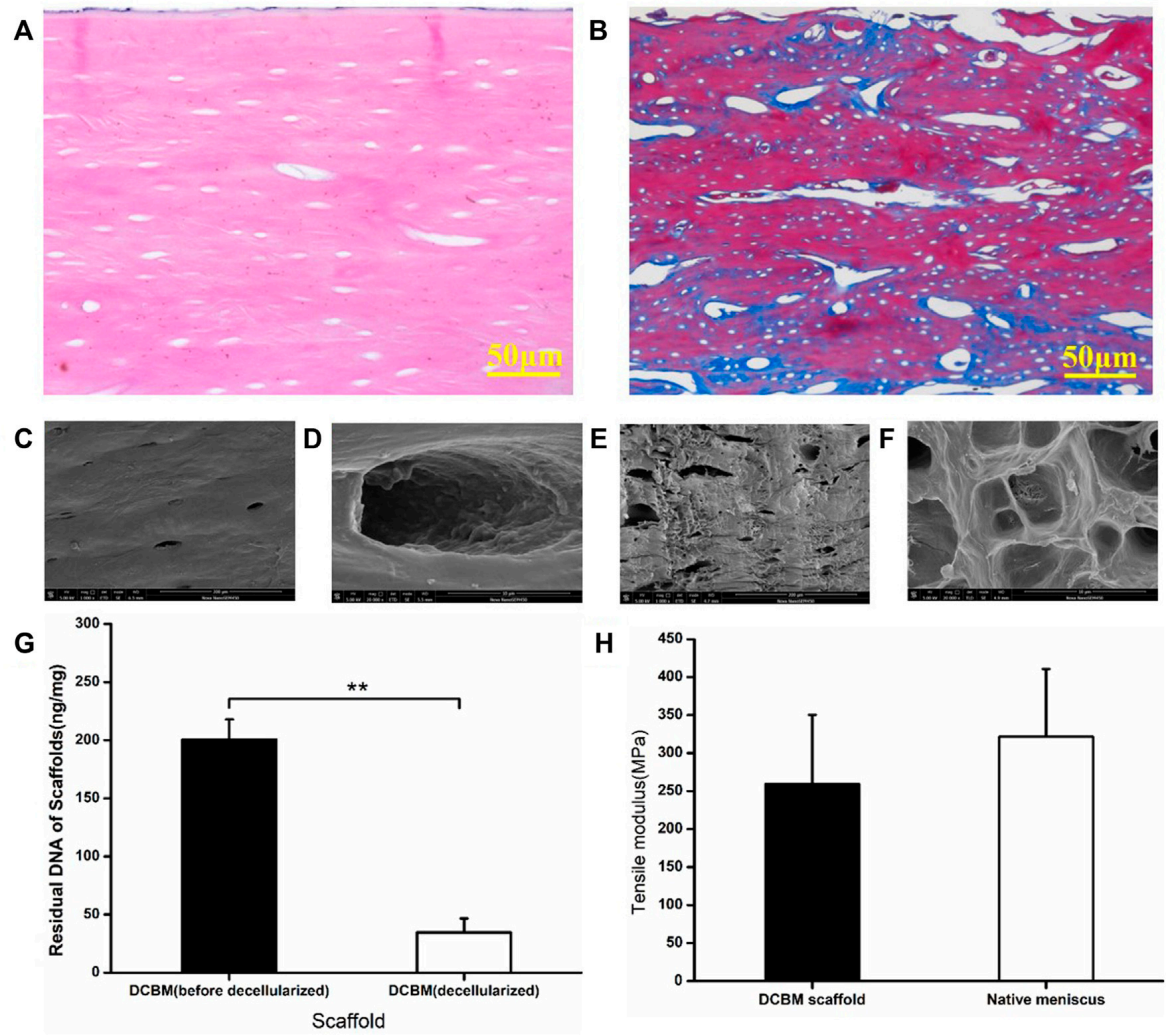
Characterization of DCBM. **(A)** HE staining showed no cells residual; **(B)** MASSON staining showed the fiber distribution of DCBM; **(C)** surface ultrastructure and **(D)** enlarge view of DCBM; **(E)** cross-section ultrastructure and **(F)** enlarge view of DCBM; **(G)** change of DNA content in DCBM after decellularization; **(H)** comparison of tensile modulus of DCBM scaffold and native meniscus. *p < 0.05.

HE staining showed no cell residues in the DCBM scaffold, and a large number of round or oval pore-like structures were retained in the material, mainly composed of hollows after decalcification and decellularization (Figure 3A). Masson staining showed similar collagen structure, void structure and distribution. The blue reaction mainly localized to the osteoid tissue and collagen fibers, and the red for lamellar bone formation (Figure 3B).

#### 3.2.2 Microstructure of the Demineralized Cortical Bone Matrix Scaffold

SEM scanning results showed that the surface of the DCBM scaffold is smooth (Figure 3C) with a few pores (Figure 3D), while the section of it is rough (Figure 3E) and full of pores (Figure 3F).

#### 3.2.3 Change of DNA Content After Decellularization

After decellularization, the DNA content of DCBM scaffold was 34.48 ± 12.14 ng/mg, which was significantly lower than before (200.75 ± 34.47 ng/mg) (*p* < 0.05) (Figure 3G).

#### 3.2.4 Biomechanical Property of the Demineralized Cortical Bone Matrix Scaffold

The tensile test results showed that the tensile modulus of DCBM was 259.41 ± 90.88 MPa, while the tensile modulus of rabbit meniscus was 321.62 ± 89.09 MPa. The tensile modulus of DCBM scaffold was slightly lower than that of the rabbit meniscus; however, the difference is not statistically significant (*p* > 0.05) (Figure 3H).

### 3.3 The Interaction of Peripheral Blood-Derived Mesenchymal Stem Cells and Demineralized Cortical Bone Matrix

#### 3.3.1 Cytotoxicity of the Demineralized Cortical Bone Matrix Scaffold

**FIGURE 4:**
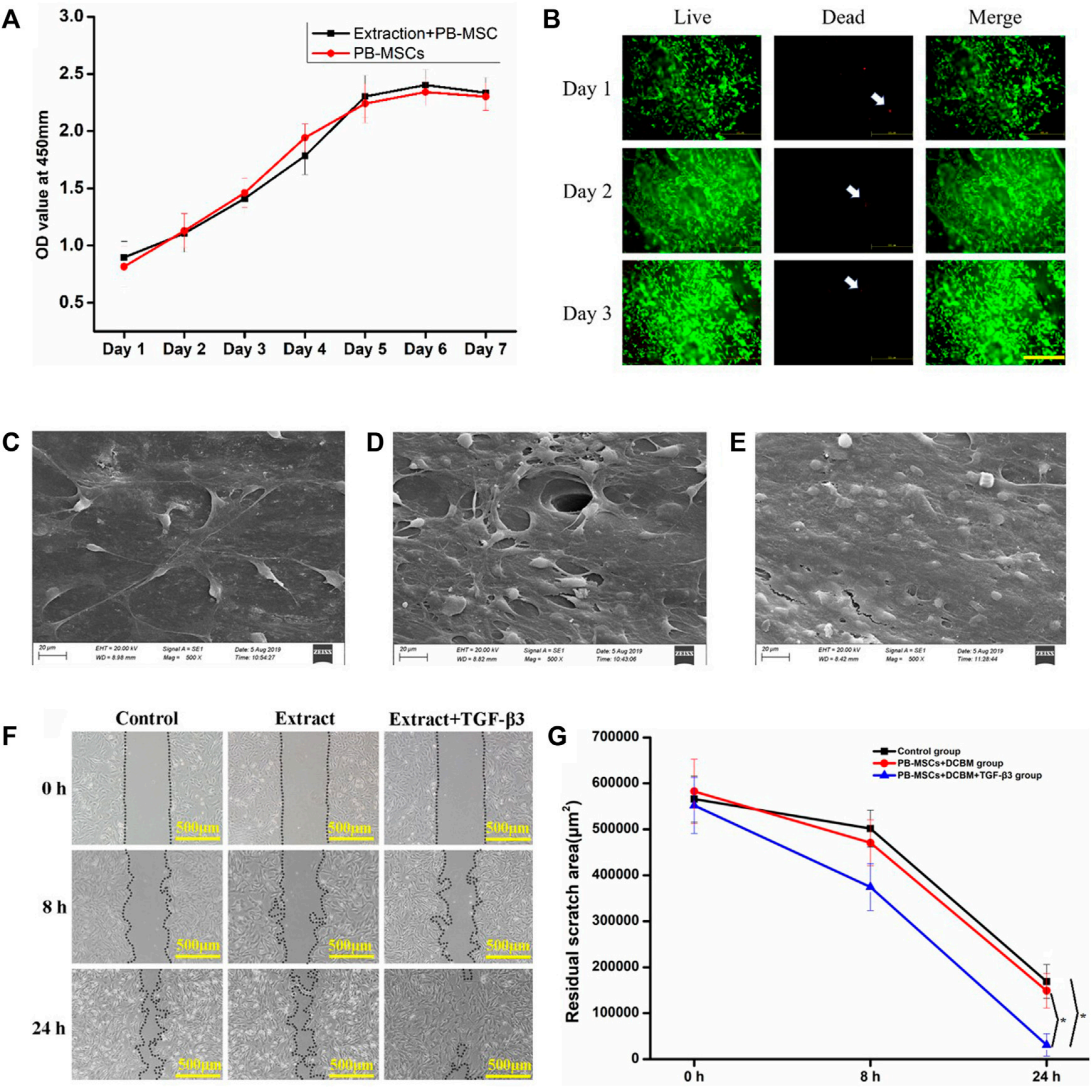
Interaction of PBMSCs and DCBM. **(A)** comparison of cell proliferation ability in DCBM extract group and control group; **(B)** Live/Dead staining analysis of PBMSCs seeded on DCBM. Green represented live cells while red represented dead cells. White arrow pointed the dead cells. Scale bar: 500 μm; the surface ultrastructure of DCBM scaffolds seeded with PBMSCs in **(C)** 1st, **(D)** 2nd and **(E)** 3rd day; **(F)** remaining area at different time points after scratching; **(G)** quantitative analysis of remaining area from different groups. *p < 0.05.

The cell viability of the two groups evaluated by CCK-8 increased with time on the first 5 days. On the 6th to 7th days, cell proliferation entered a plateau phase. Compared with the normal medium group, the DCBM extract group showed similar cell viability at every assessing timepoint (Figure 4A).

#### 3.3.2 Cytocompatibility of the Demineralized Cortical Bone Matrix Scaffold

The live/dead staining showed PBMSCs could adhere to the surface of the scaffold well, indicating good cell viability. Over time, the number of living cells has increased significantly. From 1st day to 3rd day, only a few dead cells were seen on the scaffold (Figure 4B).

#### 3.3.3 Adhesion of Peripheral Blood-Derived Mesenchymal Stem Cells

The SEM results confirmed the live/dead staining results. On 1st day, a few cells can spread and adhere to the surface of the scaffold (Figure 4C). On 2nd day, the cells on the surface of the scaffold increase significantly (Figure 4D). On 3rd day, layers of PBMSCs were on the surface of the scaffold (Figure 4E).

#### 3.3.4 Migration of Peripheral Blood-Derived Mesenchymal Stem Cells

Migration occurred in all three groups and the remaining scratch area was marked and calculated (Figure 4F). The migration speed of PBMSCs in the extract + TGF-β3 group was significantly faster than in the extract and control groups. At 24 h after the scratch, the area difference was statistically significant (*p* < 0.05). The left 2 groups showed no significant difference at each time point (*p* > 0.05) (Figure 4G). The results showed that the extract did not influence the migration of PBMSCs while the TGF-β3 promoted it.

#### 3.3.5 Chondrogenic Differentiation of Peripheral Blood-Derived Mesenchymal Stem Cells on Scaffold

##### 3.3.5.1 Evaluation of Glycosaminoglycan Content on Scaffold

**FIGURE 5:**
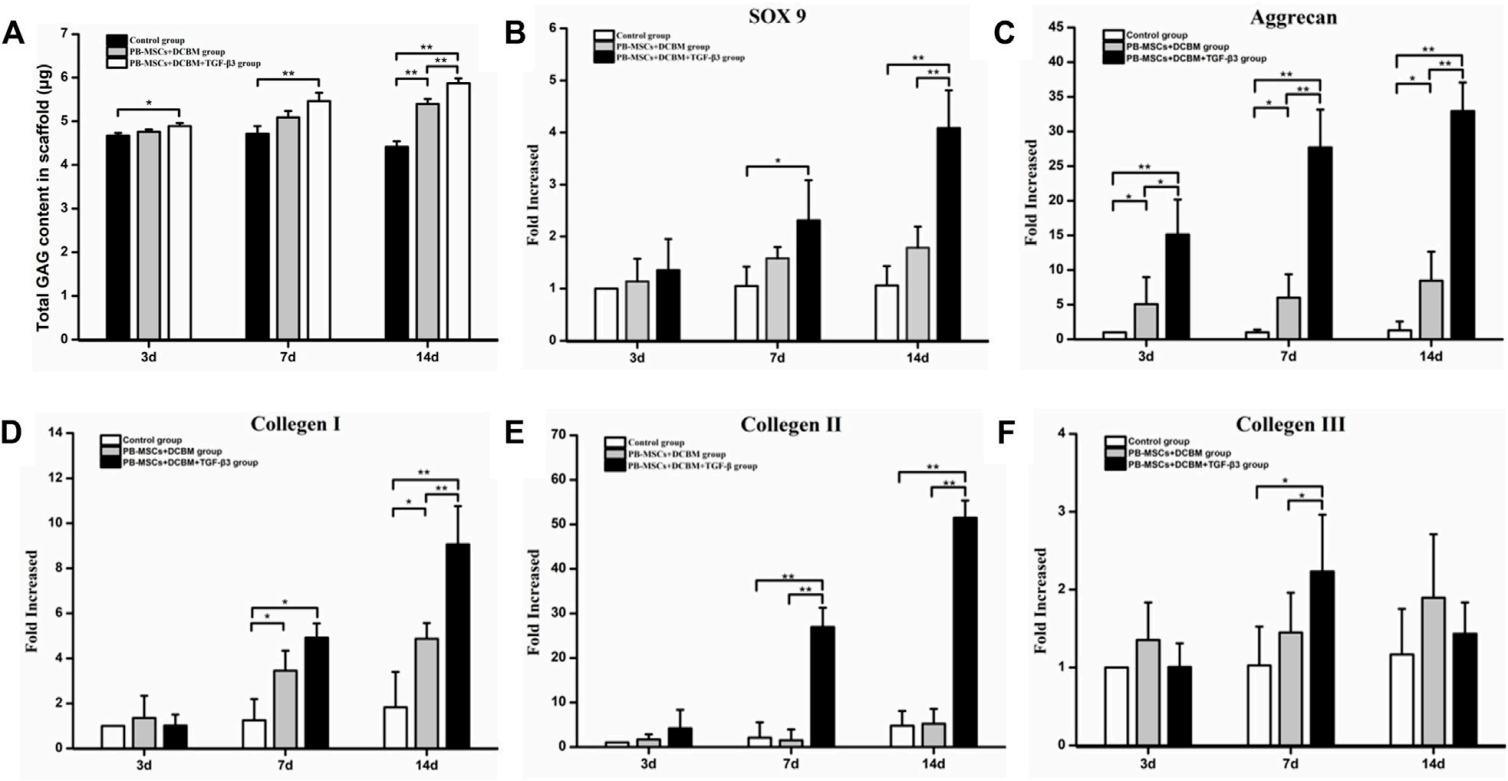
Evaluation of differentiation capacity of PBMSCs on DCBM. **(A)** comparison of GAG production of different groups; real-time PCR analysis of genes expression of **(B)** SOX 9, **(C)** Aggrecan, **(D)** collagen Ⅰ, **(E)** collagen Ⅱ and **(F)** collagen Ⅲ of the PBMSCs seeded on DCBM. *p < 0.05, **p < 0.01.

The content of GAG secreted by PBMSCs in the DCBM + TGF-β3 group was higher than that in the control group at every time point (all *p* < 0.05), while the GAG secreted by PBMSCs on the scaffold of the DCBM group was higher than that of the control group only on 14th day (*p* < 0.05) (Figure 5A).

##### 3.3.5.2 Evaluation of Cartilage-Related Gene Expression

As for SOX 9, the expression level in the DCBM + PBMSCs + TGF-β3 group was significantly higher than that in the control group on the 7th day (*p* < 0.05). On the 14th day, the expression of SOX 9 was higher than that in the DCBM + PBMSCs group (*p* < 0.05) and control group (*p* < 0.05) (Figure 5B).

The expression level of aggrecan showed that the DCBM + PBMSCs + TGF-β3 group had a higher expression level than DCBM + PBMSCs group (*p* < 0.05) and control group (*p* < 0.05), on 3rd, 7th, and 14th day. DCBM + PBMSCs group had a higher expression level than control group on 3rd, 7th, and 14th day (*p* < 0.05) (Figure 5C).

On 7th day, the expression of COL Ⅰ in the DCBM + PBMSCs + TGF-β3 group was higher than that in the control group (*p* < 0.05), and the expression of COL Ⅰ in the DCBM + PBMSCs group was higher than that in the control group (*p* < 0.05), too. On 14th day, the expression of COL Ⅰ in the DCBM + PBMSCs + TGF-β3 group was higher than that in the DCBM + PBMSCs group (*p* < 0.05) and the control group (*p* < 0.05), while the expression of COL Ⅰ in the DCBM + PBMSCs group was higher than that in the control group (*p* < 0.05) (Figure 5D).

As for COL Ⅱ, the expression level in the DCBM + PBMSCs + TGF-β3 group was higher than that in the DCBM + PBMSCs group (*p* < 0.05) and the control group (*p* < 0.05), on 7th and 14th day (Figure 5E).

Ad for COL Ⅲ, the DCBM + PBMSCs + TGF-β3 group showed a higher expression than the DCBM + PBMSCs group (*p* < 0.05) and the control group (*p* < 0.05) only on the 7th day (Figure 5F).

### 3.4 *In Vivo* Experiment

#### 3.4.1 Macroscopic Observations of Meniscus and Cartilage

At 12 weeks after the operation, the synovial limbus of the meniscus in the DCBM + PBMSCs group was closely connected with the joint capsule and the articular cartilage surface of the femoral condyle and the tibial plateau was smooth. So were the meniscus in the DCBM and sham operation groups. There was no obvious new tissue formation of the meniscus in the control group, and the cartilage surface of the femoral condyle and the tibial plateau was not smooth, with visible abrasion.

**FIGURE 6:**
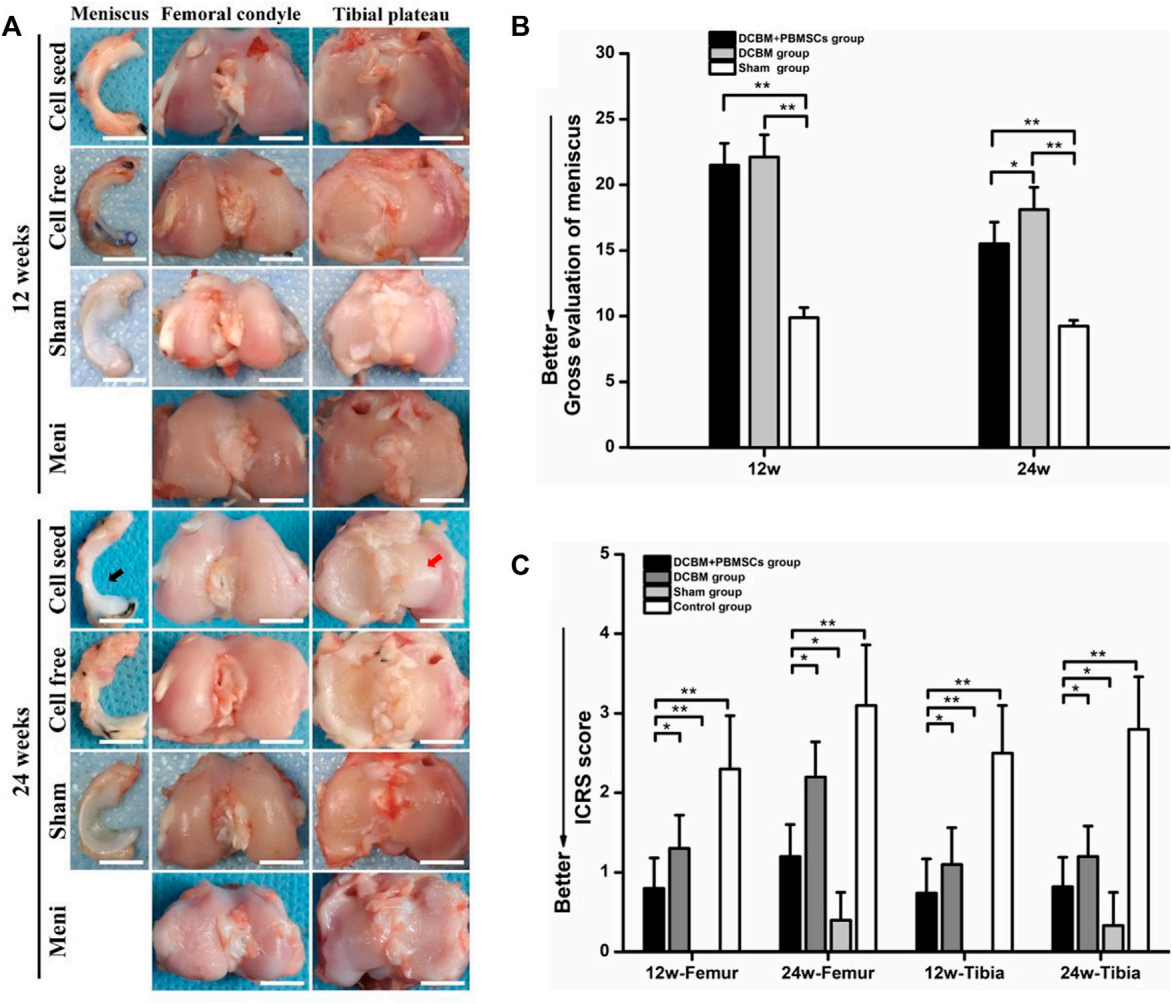
**(A)** Macroscopic observations and quantitative evaluation of **(B)** meniscus and **(C)** cartilage at 12 and 24 weeks postoperatively. Black arrow showed the DCBM + PBMSCs group gained mature meniscus which was similar to normal meniscus after 24 weeks. Red arrow showed smooth articular surface. Scale bar = 200 μm, *p < 0.05, **p < 0.01.

At 24 weeks after the operation, the new meniscus tissue in the DCBM + PBMSCs group was mature and similar to the normal meniscus. The synovial limbus was tightly connected with the joint capsule, the articular cartilage of the femoral condyle and tibial plateau was slightly torn, and no obvious osteophytes were found. In the DCBM group, the meniscus was closely connected with the joint capsule, the cartilage surface of the femoral condyle and the tibial plateau was not smooth, and a small amount of osteophyte was formed on edge. The meniscus in the sham operation group was normal meniscus shape, and the articular surfaces of the femur and tibia were normal. No new meniscus tissue was observed in the control group, and the cartilage surface of the femoral condyle and the tibial plateau was rough, with severe wear and obvious osteophytes (Figure 6A).

Quantitative evaluation of meniscus showed that at 12 weeks, the general meniscus score of the DCBM + PBMSCs group was 21.50 ± 1.66, while the score of the DCBM group was 22.13 ± 1.69. There was no statistically significant difference between the two groups (*p* > 0.05). However, scores of DCBM + PBMSCs and DCBM group were higher than those of the sham operation group (*p* < 0.05), indicating that at 12 weeks, the difference between the newly formed meniscus and the normal meniscus still existed. At 24 weeks, the scores in the DCBM + PBMSCs group and the DCBM group were 15.50 ± 1.66 and 18.13 ± 1.69, respectively. The scores of the DCBM + PBMSCs group were significantly lower than those of the DCBM group (*p* < 0.05), indicating that the newly formed meniscus in the DCBM + PBMSCs group had a better condition than that in the DCBM group (Figure 6B).

The overall ICRS scores showed that at 12 and 24 weeks after the operation, the scores in the DCBM + PBMSCs group and the DCBM group were lower than that of the control group (*p* < 0.05), but higher than that of the sham operation group (*p* < 0.05). At each time point, the DCBM + PBMSCs group had a lower score than the DCBM group (*p* < 0.05) (Figure 6C).

These results indicated that DCBM augmented with PBMSCs had a better meniscus repair and cartilage protection effect.

#### 3.4.2 Histological Evaluation of Meniscus

The HE staining of meniscus showed a small amount of chondroid-like cells in the DCBM + PBMSCs group and the DCBM group at 12 weeks after the operation, but the new collagen fibers were in a mess distribution. At 24 weeks after the operation, the meniscus fibrocartilage-like structure was formed in the DCBM + PBMSCs group, and its morphology was more similar to the native meniscus than the DCBM group.

Toluidine blue staining showed that at 12 weeks after the operation, the newly formed tissue in the DCBM + PBMSCs group was lightly colored, with no obvious difference between the medial and lateral parts. So was the DCBM group. At 24 weeks after the operation, the medial part of the tissue was stained deeply, as well as many cartilage lacuna structures were seen in the DCBM + PBMSCs group. The regional metachromaticity of the staining indicated that more cartilage-specific matrix GAGs were secreted medially. However, similar results did not appear in the DCBM group.

Immunohistochemistry of collagen I and II showed that at 12 weeks after the operation, collagen I in the DCBM + PBMSCs group and DCBM were strongly positive, and the expression of collagen II was only positive in the medial part. At 24 weeks after the operation, collagen I staining in the DCBM + PBMSCs group showed that the lateral side of the meniscus was strongly positive and the medial side was weakly positive. A similar result was not obvious in the DCBM group. The expression of collagen II in the medial part of the meniscus was stronger in the DCBM + PBMSCs group than in the DCBM group, and the expression of collagen II in the lateral part was negative in both groups.

**FIGURE 7:**
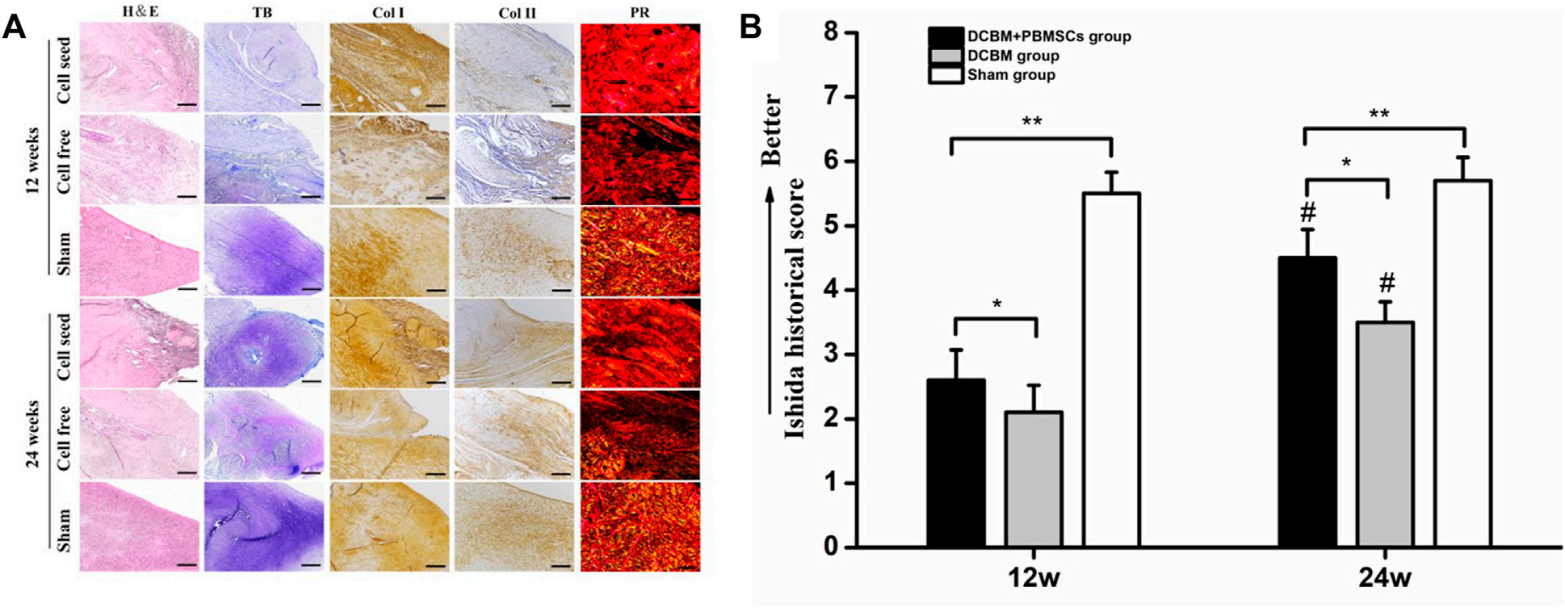
**(A)** Histological staining and **(B)** quantitative analysis of regenerated meniscus. Scale bars = 100 μm, *p < 0.05, **p < 0.01, #vs. results in 12 weeks *p* < 0.01.

The results of Sirius red staining showed that at 12 weeks after the operation, strong red or yellow refraction (collagen I fibers) and a small amount of green refraction (collagen III fibers) were seen in the newly formed meniscus in the DCBM + PBMSCs group. The DCBM group showed strong red refraction (collagen I fibers). At 24 weeks after the operation, the DCBM + PBMSCs group showed stronger refraction and the distribution of collagen fibers was similar to that of the normal meniscus. The DCBM group showed moderate-strength collagen I fibers refraction, while the distribution of collagen fibers was different from the fibrous arrangement of the normal meniscus (Figure 7A).

The Ishida score showed that the repair of meniscus in the DCBM + PBMSCs group was better than that in the DCBM group in both 12 (*p* < 0.05) and 24 weeks (*p* < 0.05). In addition, in both groups, the repair effect was better in 24 weeks than 12 weeks (*p* < 0.05). Ishida score in DCBM + PBMSCs group had no statistic difference with sham-opperated group in 24 weeks (Figure 7B).

#### 3.4.3 Histological Evaluation of Cartilage

HE staining of cartilage showed that there was no obvious cartilage degeneration at 12 weeks after the operation in the sham-operated group, the DCBM + PBMSCs group, and the DCBM group. In the DCBM group, a small number of chondrocytes were enlarged in morphology. In the meniscectomy group, the cartilage degenerated slightly, and the cartilage surface was uneven and cracked. At 24 weeks after the operation, the cartilage structure and cartilage thickness in the DCBM + PBMSCs group were nearly the same as those in the sham-operated group. The cartilage in the DCBM group and the meniscectomy group had different degrees of degeneration.

The results of toluidine blue staining showed that the DCBM + PBMSCs group and DCBM group were similar to the sham operation group at 12 weeks after the operation. The staining of the cartilage surface in the meniscectomy group became lighter, indicating that the cartilage matrix was damaged. At 24 weeks after the operation, the surface of the cartilage in the DCBM + PBMSCs group was slightly lighter, and the coloration of the cartilage in the DCBM group was significantly lighter.

**FIGURE 8:**
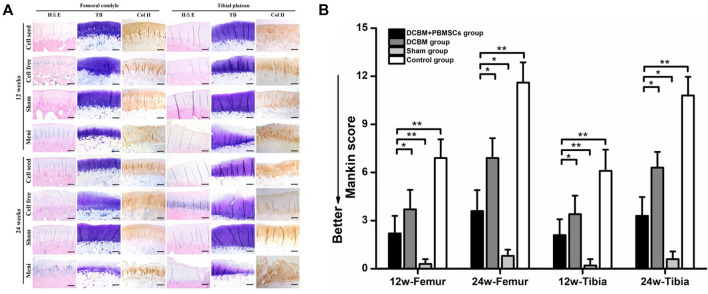
**(A)** Histological staining and **(B)** mankin score of articular cartilage. Scale bars = 100 μm, *p < 0.05, **p < 0.01.

The results of collagen Ⅱ staining showed that at 12 weeks after the operation, only the positive staining in the control group was unevenly distributed, and the surface layer was weakly positive. At 24 weeks after surgery, the results of the DCBM + PBMSCs group were similar to the sham-operated group, and both were uniformly positive. Cartilage in the DCBM group showed weak positivity with uneven distribution. The meniscectomy group had a negative result, and the articular cartilage thickness was significantly reduced (Figure 8A).

The Mankin score of cartilage histology results also showed that on 12 and 24 weeks after the operation, the femoral and tibia cartilage degeneration in the DCBM + PBMSCs group was more serious than that in the sham operation group (*p* < 0.05). However, it was significantly better than that in control group (*p* < 0.05) and DCBM group (*p* < 0.05).

## 4 Discussion

Given the limited treatment method of the meniscus, the difficulty in collecting seed cells and unsatisfied characteristics of DBM, the present study aimed to use DCBM augmented with PBMSCs to repair the injured meniscus.

The present study extracted and identified rabbit PBMSCs. Similar to our previous study in which PBMSCs were used to repair bone and cartilage, according to the positive results of histological staining, PBMSCs were proved to possess the tri-lineage differentiation ability (Fu et al., 2014; Fu et al., 2015). Combined with the results of culture and flow cytometry, according to the identify criteria of the International Society for Cellular Therapy, the cells were proved to be PBMSCs(Dominici et al., 2006).

Seed cells play an important role in tissue engineering the meniscus. In some previous studies, engineering meniscus, embryonic stem cells, BMSCs, SMSCs, and MFCs have been used and achieved some treatment effects (Baker et al., 2009; Shen et al., 2013; Ozeki et al., 2015; Yuan et al., 2017; Zhang et al., 2017). As the functional cells of the meniscus, MFCs can produce specific ECM without induction, thus are considered the optimal choice in tissue engineering meniscus (Fox et al., 2015). However, poor sources limit its application. Stem cells have a wide range of sources, which overcomes the shortcomings of MFCs to some degree. The strong proliferation and multi-directional differentiation ability also contribute to MSCs becoming the most suitable seed cells, of which BMSCs, ADSCs and SMSCs were mostly used. However, the above-mentioned MSCs faced the shortcomings of traumatic extraction methods, the need for anesthesia, and the difficulty of repeated access (De Bari et al., 2001; Shirasawa et al., 2006; Nerurkar et al., 2011). Compared to them, PBMSCs were easy to collect. As a type of MSC, PBMSCs were able to differentiate and afford the function of seed cells. Therefore, it was selected as the seed cells to repair the meniscus in the present study.

Natural materials or ECM components have good biological activity, high biomimetic properties, and good biocompatibility and are widely used in tissues or organs in tissue engineering (Chen et al., 2017; Ruprecht et al., 2019; Stein et al., 2019). Demineralized bone matrix (DBM), as a natural tissue-derived scaffold material, is widely used in bone tissue engineering (Zhang et al., 2019; Cho et al., 2020). In recent years, studies have also been using DBM for soft tissue repair (Yamada, 2004). There have been studies using the allogeneic decalcified cancellous bone matrix as a scaffold material to repair the meniscus, but the cancellous bone has the disadvantages of weak biomechanical strength and difficult fixation (Arnoczky et al., 2010). Compared to decalcified cancellous, DCBM possess superior biomechanics. Therefore, the present study used DCBM as the scaffold material for tissue engineering meniscus. Moreover, the compression modulus, which was not inferior to the native meniscus supported DCBM as a scaffold for meniscal repair.

The histological staining and DNA content test showed that we effectively removed the cellular components, as well as reduced the DNA content of the cortical bone. The cells or cell fragments in the scaffold may induce an immune response, aggravate the inflammatory response, cause severe pain, and slow down the process of repair (Zheng et al., 2005; Dong et al., 2015). In addition, excessive DNA content in the material can also cause an immune response in the body; thus, the degradation rate of the stent material is accelerated, which increases the failure rate of the stent material. Studies have found that when the DNA content in the material is less than 50 ng/mg, or the DNA fragment is less than 200 bp, it can greatly reduce the immune response and promote the tissue remodeling of the scaffold at the appropriate site. In other words, a decellularization procedure is necessary and can effectively reduce or remove the immunogenicity of natural tissue scaffold materials (Branch, 2011). In this experiment, the HE staining confirmed almost no residual cells in the decellularized DCBM. Moreover, after DNase and RNase treatment, the DNA content measurement results of the material showed that the DNA content in the DCBM scaffold material is less than 50 ng/mg, which met the requirements.

After demonstrating the absence of cytotoxicity of DCBM by CCK8, live-dead staining and scanning electron microscopy, we further evaluated the chondrogenic capacity of PBMSCs on the material. As one of the most famous chondrogenesis-promoting cytokines, TGF-β3 was chosen to induce the chondrogenesis of PBMSCs(Janssen et al., 2019; Hashemibeni et al., 2021). GAG assay results on the 14th day showed that PBMSCs in the PBMSCs + DCBM + TGF-β3 group secreted most GAGs among 3 groups, and PBMSCs + DCBM had a better result than control. The results confirmed the effect of TGF-β3 and indicated that DCBM might have the chondrogenesis-inducing ability. However, whether the chondrogenesis-inducing ability of DCBM is effective or not *in vivo* is to be confirmed.

In order to verify the effect of DCBM scaffold materials on chondrocytes differentiation in gene level, the present study evaluated the expression of SOX9, ACAN, COL I, COL II, and COL III. The results showed that the DCBM + PBMSCs + TGF-β3 group promoted the expression of fibrocartilage-related genes SOX 9, ACAN, COL Ⅰ, and COL Ⅱ to varying degrees. Among these genes, the expression of SOX 9 is the strongest indicator of cartilage formation. SOX 9 is a key transcription factor that plays a role in differentiating PBMSCs into fibrochondrocytes (Liang et al., 2018). Therefore, we chose SOX 9 as a marker for stem cell chondrogenic differentiation in this experiment. The expression of SOX 9 in the DCBM + PBMSCs + TGF-β3 group was 2.1 and 3.9 times higher than that of the control group at 7 and 14 days, respectively. Moreover, there was no significant difference in SOX 9 expression between the DCBM + PBMSCs group and the control group, which indicated that the scaffold was difficult to induce the differentiation of PBMSCs into chondrocytes. However, the addition of TGF-β3 promotes the differentiation of PBMSCs into cartilage. It is well known that TGF-β stimulates MSCs cartilage formation promotes mesenchymal coagulation and enhances the production of cartilage ECM (Wang et al., 2014). Among them, TGF-β3 is one of the main signal cascades for chondrogenic differentiation, which can enhance chondrocytes as well as meniscus cartilage formation (van der Kraan, 2017). The expression of gene ACAN showed that the expression of ACAN was higher than the other two groups on 3, 7 and 14 days (*p* < 0.05), and the DCBM + PBMSCs group was also higher than the control group (*p* < 0.05), but the expression did not increase with time. Consistent with previous reports, the presence of TGF-β3 significantly promotes stem cell ACAN expression. As the main component of the extracellular matrix of chondrocytes, the expression of ACAN is related to the accumulation of cartilage-like substances (Ozeki et al., 2021). As for the gene COL Ⅰ, the expression of COL Ⅰ in the DCBM + PBMSCs + TGF-β3 group was higher than the other two groups on the 7th and 14th day (*p* < 0.05), and the DCBM + PBMSCs group was also higher than the control group (*p* < 0.05). Since DCBM itself is mainly type I collagen, it promotes the expression of COL I to a certain extent, and the microenvironment of the scaffold is suitable for meniscus regeneration. The expression level of gene COL Ⅱ at 7 and 14 days was higher than that of the other two groups (*p* < 0.05), but there was no statistical significance between the DCBM group and the control group (*p* > 0.05). Compared with the control group, the DCBM scaffold group promoted the expression of genes ACAN and COL I, but did not significantly promote the expression of COL II and COL Ⅲ, indicating that the DCBM scaffold material alone has limited fibrocartilage induction. Moreover, the expression of COL Ⅲ in DCBM + PBMSCs + TGF-β3 was higher than the other two groups only on the 7th day (*p* < 0.05), as time went by, it also rose first then fell. The study of Wang et al. (2020) showed that type III collagen has a potential regulatory role in the early stages of type II collagen fiber formation and chondrocyte mechanical transduction (Wang et al., 2020). This experiment shows that under the stimulation of TGF-β3, the early expression of the gene COL Ⅲ is promoted to a certain extent.

The *in vivo* study confirmed the *in vitro* study that PBMSCs/DCBM scaffold pretreated with TGF-β3 could repair the meniscus injury. In addition, the repaired group possessed a better outcome in the cartilage of the femur and tibia than a control group, which indicated PBMSCs/DCBM could protect the cartilage and relieve osteoarthritis. Some previous studies have also come up with results on this topic. A recent study indicated that the PCL/SF/Gel/AA composite scaffolds seeded with allogeneic ASCs could successfully improve meniscus healing in damaged rabbits (Abpeikar et al., 2021). However, synthetic materials risk degradation-related toxicity, stress shielding, changes in cellular phenotype, and tissue remodeling (Lee et al., 2017). Moreover, the limited follow-up time and lack of histological studies were less persuasive. Another study used PBMSCs with a polyurethane scaffold to repair a meniscus tear and found PBMSCs did not show any advantage in protecting articular cartilage over acellular scaffolds (Olivos-Meza et al., 2021). However, the clinical study only used MRI as the primary assessment, and the small number of patients, lack of randomization and deficiency of histologic examination also made them less persuasive. In addition, though there are clinical studies in treating meniscus injury with PBMSCs, few basic studies use PBMSCs.

## 5 Conclusion

The DCBM scaffold has excellent biomechanical properties and cell compatibility and is a reliable tissue engineering meniscus material. In the meniscus reconstruction, PBMSCs can differentiate and assume the function of seed cells. The DCBM/PBMSCs complex pretreated with TGF-β3 can better promote the repair and regeneration of the rabbit meniscus, protect the cartilage of the knee joint, and slow down the process of joint degeneration.

## Data Availability

The raw data supporting the conclusions of this article will be made available by the authors, without undue reservation.
